# InTxDB: interaction data between gram-negative bacteria secreted effectors and host proteins

**DOI:** 10.1093/database/baaf070

**Published:** 2025-10-31

**Authors:** Yanyan Zhu, Liya Liu, Yueming Hu, Sida Li, Enyan Liu, Yanshi Hu, Shilong Zhang, Haoyu Chao, Qiuyu Fang, Huan Yu, Ming Chen

**Affiliations:** Department of Bioinformatics, College of Life Sciences, Zhejiang University, 866 Yuhangtang Road, Xihu District, Hangzhou 310058, China; Department of Bioinformatics, College of Life Sciences, Zhejiang University, 866 Yuhangtang Road, Xihu District, Hangzhou 310058, China; Department of Bioinformatics, College of Life Sciences, Zhejiang University, 866 Yuhangtang Road, Xihu District, Hangzhou 310058, China; Anhui Province Key Laboratory of Embryo Development and Reproductive Regulation, School of Biological and Food Engineering, Fuyang Normal University, 100 Qinghe West Road, Fuyang 236037, China; Department of Bioinformatics, College of Life Sciences, Zhejiang University, 866 Yuhangtang Road, Xihu District, Hangzhou 310058, China; Department of Bioinformatics, College of Life Sciences, Zhejiang University, 866 Yuhangtang Road, Xihu District, Hangzhou 310058, China; Department of Bioinformatics, College of Life Sciences, Zhejiang University, 866 Yuhangtang Road, Xihu District, Hangzhou 310058, China; Department of Bioinformatics, College of Life Sciences, Zhejiang University, 866 Yuhangtang Road, Xihu District, Hangzhou 310058, China; Department of Bioinformatics, College of Life Sciences, Zhejiang University, 866 Yuhangtang Road, Xihu District, Hangzhou 310058, China; Department of Thoracic Surgery, The Second Affiliated Hospital Zhejiang University School of Medicine, Zhejiang University, No. 88 Jiefang Road, Shangcheng District, Hangzhou 310009, China; Department of Thoracic Surgery, The Second Affiliated Hospital Zhejiang University School of Medicine, Zhejiang University, No. 88 Jiefang Road, Shangcheng District, Hangzhou 310009, China; Department of Bioinformatics, College of Life Sciences, Zhejiang University, 866 Yuhangtang Road, Xihu District, Hangzhou 310058, China; Zhejiang Key Laboratory of Multi-omics Precision Diagnosis and Treatment of Liver Diseases, The Second Affiliated Hospital Zhejiang University School of Medicine, Zhejiang University, No. 88 Jiefang Road, Shangcheng District, Hangzhou 310009, China; Bioinformatics Center, The First Affiliated Hospital Zhejiang University School of Medicine, Zhejiang University, No. 79 Qingchun Road, Shangcheng District, Hangzhou 310003, China

## Abstract

Gram-negative bacteria utilize specialized secretion systems to deliver effectors into the cytoplasm of eukaryotic cells, where they manipulate host cellular functions to promote infection. These interactions play a crucial role in bacterial pathogenesis and pose significant public health challenges. Understanding of the protein–protein interactions (PPIs) between effectors and host proteins is essential for deciphering infection mechanisms and developing novel therapeutic strategies. However, existing databases on pathogen–host interactions often lack comprehensive coverage of secreted effectors and their host targets, limiting their utility in studying bacterial infections. Furthermore, current resources are often incomplete, poorly annotated, and lack predictive capabilities. To address these limitations, we have developed InTxDB, a comprehensive database dedicated to interactions involving bacterial type I-X secreted effectors and their host counterparts. InTxDB currently includes 1829 experimentally validated interaction pairs from 100 bacterial species, with detailed annotations on protein sequences, functions, subcellular localizations, secretion signals, local structural and functional properties, structures, and interaction sites. By filling a critical gap in existing databases, InTxDB provides a well-curated and functionally enriched resource to advance research on bacterial pathogenesis and host–pathogen interactions.

## Introduction

Gram-negative bacteria utilize specialized secretion systems to transport proteins across their cell membranes, with at least 10 distinct systems identified [1–3]. These secretion systems can be classified into two main types. The first type, such as the type III secretion system (T3SS), enables the direct translocation of substrate proteins into eukaryotic host cells in a single step [4]. The second type, including the Sec and Tat pathways, initially transports substrate proteins to the periplasm, followed by a secondary mechanism that transfer across the outer membrane or into the host cell [5]. The translocated substrate proteins, known as effectors, play a critical role in bacterial infection and pathogenicity by manipulating host cellular processes to promote survival and proliferation [1, 5].

Once translocated into the host cytoplasm, bacterial effectors disrupt essential signalling pathways, leading to disease. For example, the T3SS effector YopJ, secreted by *Yersinia pestis*, inhibits the MAPK and NF-κB signalling pathways, suppressing the host inflammatory response and allowing immune detection [6]. The T4SS effector CagA, produced by *Helicobacter pylori*, binds to the SH2 domain of host proteins, activating SHP-2 phosphatase and inducing sustained ERK/MAPK signalling pathway, which promotes abnormal gastric epithelial cell proliferation and contributes to gastric cancer development [7]. Additionally, the T6SS effector VgrG, secreted by *Pseudomonas aeruginosa*, promotes inflammasome assembly and activates the NLRP3 signalling pathway, triggering the release of pro-inflammatory cytokines such as IL-1β, leading to a strong inflammatory response [8].

Over the past few decades, experimental techniques such as co-immunoprecipitation (Co-IP) and high-throughput methods like mass spectrometry (MS) and yeast two-hybrid (Y2H) [9–11] have significantly expanded our understanding of protein–protein interactions (PPIs) between gram-negative bacterial effectors and host proteins. These techniques curated in several host–pathogen protein interaction databases [12–14]. For example, PHISTO integrates host–pathogen PPIs and provides function annotations of pathogen-targeted human proteins [12]. STRING offers extensive PPI datasets with confidence score to assess interaction reliability [13]. IntAct provides detailed biological context and functional annotations for each interaction [14]. PHI-base specializes in pathogen–host interactions with documented phenotypic outcomes [15]. HoPaCI-DB focuses specifically on *Pseudomonas* and *Coxiella* effector systems [16], while DBSecSys focuses on *Burkholderia* effectors [17]. For secretion system effectors, dedicated databases exist including BastionHub for T1SE–T4SE and T6SE [18], T3DB for type III secretion systems [19], and SecretoMyc for mycobacterial effectors [20]. However, current databases collectively lack comprehensive coverage of all bacterial secretion system types (T1SE–T10SE), and they often lack atomic-level details, such as binding sites and structural information (Table 1).

**Table 1.** Comparative analysis of major host–pathogen interaction and secretion system databases

Database	Scope of interactions	Method	Structural data	Site information	Subcellular localization	Interaction network	Predicted interfaces
PHISTO	Pathogen–host	Experimental data	No	No	No	No	No
HPIDB	Pathogen–host	Experimental data	No	No	No	No	No
MINT	Pathogen–host	Experimental data	No	No	No	No	No
PHI-base	Pathogen–host	Experimental data	No	No	No	No	No
IntAct	Pathogen–host	Experimental data	No	Yes	No	Yes	No
HoPaCI-db	*Pseudomonas aeruginosa* and *Coxiella burnetiid –*host	Experimental data	No	No	Yes	Yes	No
BastionHub	T1SE–T4SE, 6SE	Experimental data	No	No	No	No	Yes
T3DB	T3SE	Experimental data	No	No	No	No	Yes
SecretoMyc	Mycobacterial effectors	Experimental data	No	No	No	No	No
DBSecSys	*Burkholderia mallei–*host and *Burkholderia pseudomalleil*–host	Experimental data and predicted data	No	No	No	No	No

To address this gap, we have developed InTxDB (http://bis.zju.edu.cn/InTxDB), a dedicated database for gram-negative bacteria type I-X secreted effectors and their host interactions. InTxDB currently contains 1829 experimentally validated interaction pairs from 100 bacterial species, with comprehensive annotations on protein sequences, functions, subcellular localizations, secretion signals, local structural and functional properties, structures, and interaction sites. By providing a well-curated and functionally enriched dataset, InTxDB serves as a valuable resource for studying bacterial pathogenesis and host–pathogen interactions, facilitating further research and therapeutic development.

## Materials and methods

### Collection of experimentally verified effector-human PPI data

**Figure 1.**
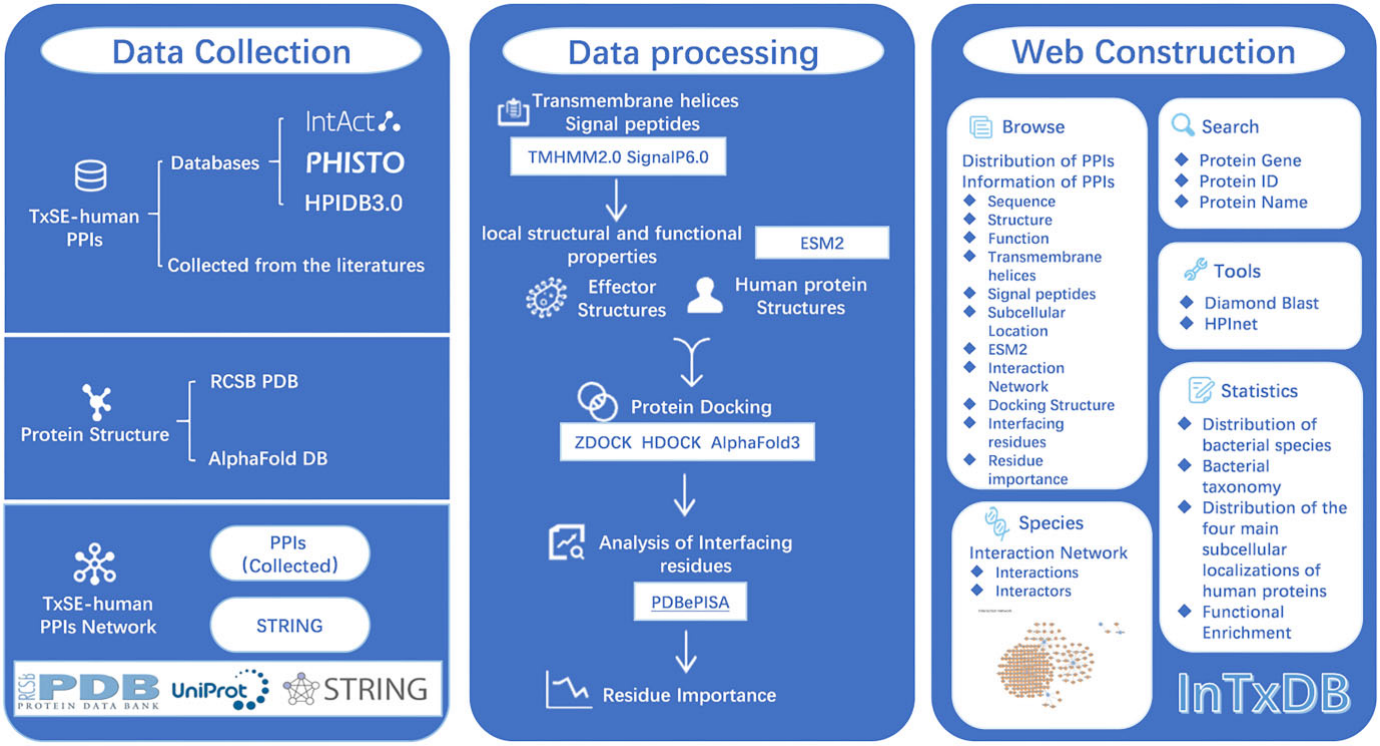
The workflow of InTxDB. The construction of InTxDB involves three main stages: data collection, data processing, and web construction. Data collection integrates three components: TxSE-human PPIs from databases and literature, structural data from PDB and AlphaFold DB, and interaction networks derived from compiled PPIs and STRING. Data processing includes transmembrane helix, signal peptide prediction (TMHMM2.0, SignalP6.0) and analysis of protein’s local structural and functional properties(ESM2), protein docking simulations (ZDOCK, HDOCK, AlphaFold3), and interaction site analysis (PDBePISA). The web platform offers an interactive interface with advanced browsing, search, and computational analysis tools for predicting and studying bacterial effector–host interactions, serving as a comprehensive resource for researchers.

We collected experimentally validated PPIs from three public databases: HPIDB [21], PHISTO [12], and IntAct [14], as well as recently published literature (Fig. 1). First, we obtained experimentally validated effectors from two public databases: SecReT4 [22] and TxSEdb (http://61.160.194.165:3080/TxSEdb/,unpublished data). Next, the gene names of these effectors and their corresponding bacterial species were used as search terms to retrieve relevant PPI-related literature. Candidate articles from the search results were manually screened and curated, leading to the final collection of experimentally verified PPIs.

### Three-dimensional complex structures of effector-human PPIs

The three-dimensional structures of bacterial effectors and human proteins were sourced from the Protein Data Bank (PDB) [23] databases and AlphaFold [24] databases (Fig. 1). Selection criteria mandated that the protein structures must contain full-length sequences. In cases where the required structures were unavailable in the PDB databases, they were obtained from the AlphaFold database.

The three-dimensional structures of effector-human PPIs were generated through docking simulations using three software tools: ZDOCK [25], HDOCK [26], and AlphaFold3 [27]. ZDOCK and HDOCK performed docking using PDB files, whereas AlphaFold3 utilized protein sequences. Protein sequences were retrieved from UniProt [28] and NCBI [29] databases. For proteins with sequences exceeding 1500 amino acids in length, only the first 1500 amino acids were selected for docking. This truncation strategy, while ensuring computational feasibility, inevitably introduces certain limitations: potential interaction sites located in the C-terminal regions of very large proteins may be omitted, leading to incomplete structural coverage and possibly underestimating biologically relevant binding events. As such, these results should be interpreted with caution, and future updates of our database will consider domain-based [30] or overlapping-fragment docking [31] strategies to mitigate this limitation.

### Prediction of effector transmembrane helices, signalling peptides, and subcellular localization

Transmembrane helices were predicted using TMHMM 2.0 [32] software, and signal peptides along with their cleavage sites were predicted using SignalP 6.0 [33] software. Subcellular localization data were retrieved from the UniProt database. For proteins with unknown subcellular localization, predictions were generated using PSORTb v3.0 [34] and DeepLocPro [35].

### Interaction interface analysis

Interfacing residues are predicted using PDBePISA [36]. Residue importance was analysed using PDBePISA, and the BURIEDSURFACEAREASCORE values were normalized by first subtracting the mean from each value and then dividing by the standard deviation.

### Network construction

STRING (https://string-db.org/) [13] is a database that systematically collects and integrates PPIs, including physical interactions and functional associations. All interaction networks associated with the specified protein were downloaded from the STRING database by selecting the protein species and inputting the protein sequence or protein name. The user can freely set the confidence level to select the desired interaction network. The resulting interaction dataset was exported in a compatible TSV format for further organization and network integration.

### Analysis of protein’s local structural and functional properties

We integrated the ESM2 model [37] to compute per-residue embeddings across each protein sequence. These embeddings were generated using the ESM2-large model [37], producing a 1280-dimensional vector for every amino acid position. The resulting values are visualized as an interactive plot directly on individual protein pages, where users can dynamically explore regions of high or low embedding magnitude corresponding to putative functional domains, conserved sites, or disordered regions.

### Database construction

InTxDB utilizes Django as the backend web framework. A MySQL service is employed to store data tables, including the basic information of bacterial effectors and host PPI entries. Network visualization is interactively presented using Cytoscape.js [38] and ECharts.js [39] plugins. The Mol* (/'molstar/) [40], a modern web-based open-source tool, is used for the visualization and analysis of large-scale molecular data, particularly for displaying three-dimensional protein structures.

## Results

### Overall description of InTxDB

InTxDB is a comprehensive database dedicated to interactions between gram-negative bacterial type I-X secreted effectors (TxSEs) and their human host proteins. It currently contains 1829 experimentally validated interaction pairs from 100 bacterial species (Table 2). Each entry is extensively annotated with information on protein sequences, functions, subcellular localizations, secretion signals, local structural and functional properties, structural data, and interaction sites.

**Table 2.** The number of PPIs and the number of bacteria effectors, bacterial species, and human proteins of each interaction

Type	Number of bacterial effectors	Number of human protein	Number of bacterial species	Number of PPIs
T1SE	11	24	16	40
T2SE	4	5	3	6
T3SE	58	767	16	1228
T4SE	58	276	24	414
T5SE	30	37	28	62
T6SE	3	10	1	10
T7SE	11	33	11	38
T8SE	4	2	3	5
T9SE	5	6	3	8
T10SE	3	11	3	18

The database architecture consists of three main components: data collection, data processing, and web construction (Fig. 1). Data collection involves extracting experimentally validated PPIs from literature and databases such as IntAct [14], PHISTO [12], and HPIDB3.0 [21], while protein structures are sourced from RCSB PDB [23] and AlphaFold DB [24]. Data processing includes transmembrane helix and signal peptide prediction (TMHMM2.0 [32], SignalP6.0 [33]), analysis of protein’s local structural and functional properties (ESM2 [37]), protein docking simulations (ZDOCK [25], HDOCK [26], AlphaFold3 [27]), and interaction site analysis (PDBePISA [36]). Finally, in the web construction phase, users can explore interaction data through a searchable and interactive interface, with tools such as Diamond [41] and HPInet [42], alongside statistical insights into bacterial species, taxonomy, and interaction distributions.

By integrating high-confidence experimental data, structural predictions, and computational tools, InTxDB serves as a valuable resource for studying bacterial pathogenesis and host–pathogen interactions.

### User interface of InTxDB

**Figure 2.**
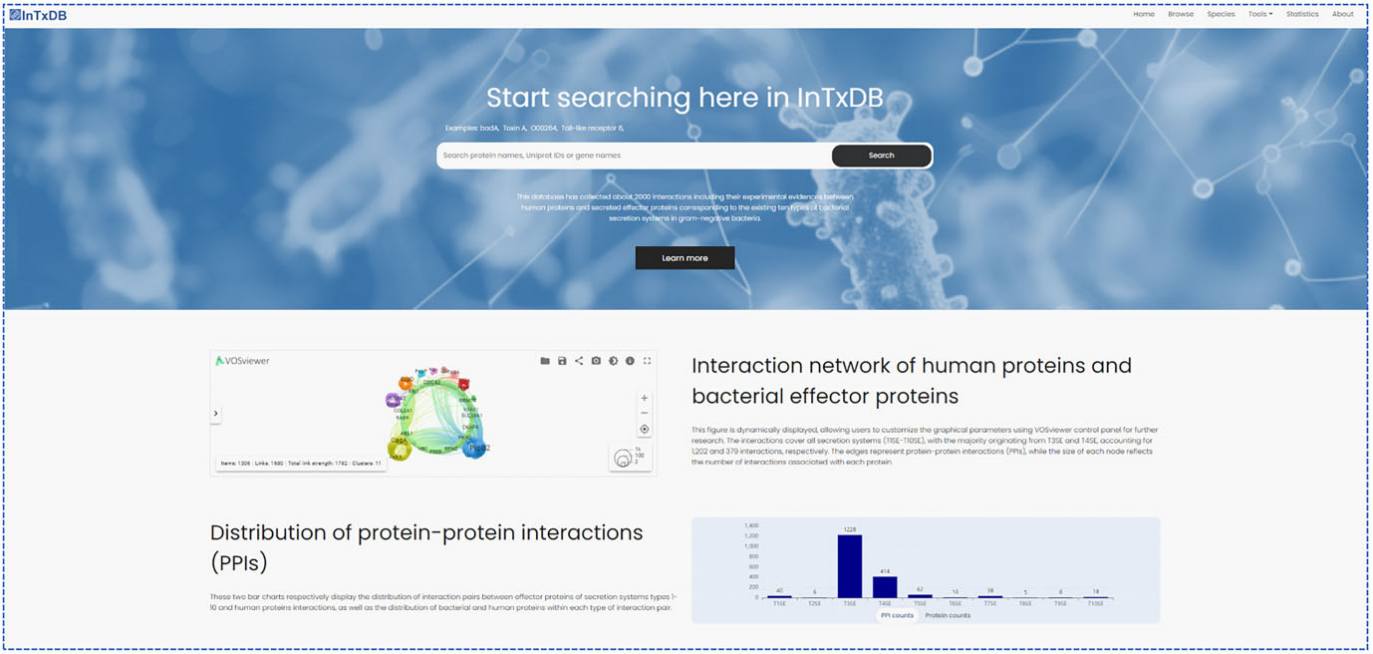
InTxDB database home page and statistics of datasets.

InTxDB provides a user-friendly interface for exploring effector proteins and their interactions through multiple search options. Figure 2 illustrates the home page, which features a quick search bar, allowing users to retrieve proteins using gene names, protein names, or protein IDs. Additionally, the home page presents statistical summaries of effectors and their interactions, along with a visual representation of the interaction network, offering an intuitive overview of the database’s content and connectivity.

### Browse and data exploration

**Figure 3.**
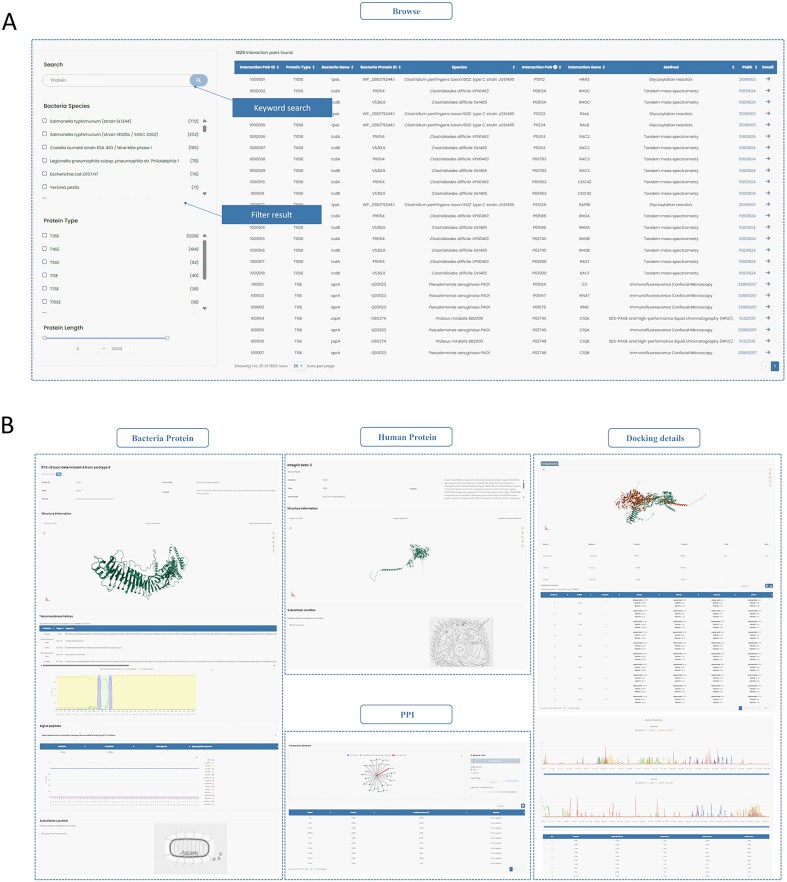
Two modules of InTxDB. (A) Browse module: Users can filter interaction data using options such as bacterial species, secretion system type, and protein length to quickly locate relevant information. (B) Detailed information page: Displays comprehensive details on selected interactions, including protein sequences, structures, interaction networks, and docking predictions, providing insights into bacterial effector–host interactions.

InTxDB offers a user-friendly interface with an intuitive filtering system. Users can refine search results using filters on the left sidebar, including bacterial species, secretion system type, and protein length, enabling quick access to relevant information (Fig. 3A).

Upon selecting an entry, users are directed to a detailed information page, organized into four main sections: Bacterial Protein, Human Protein, PPI (Protein–Protein Interaction), and Docking Details (Fig. 3B). These sections can be easily accessed via the sidebar navigation:

**Bacterial Protein and Human Protein sections**: Display fundamental details, including protein structures. The Bacterial Protein section provides additional annotations such as signal peptides and transmembrane helices.
**PPI section**: Presents the interaction network, offering insights into the relationships between effectors and human proteins, along with detailed information for each node.
**Docking Details section**: Showcases predicted docking generated by ZDOCK [25], HDOCK [26], and AlphaFold3 [27], along with interaction site analysis from PDBePISA [36] and an assessment of residue importance for both interacting proteins.

### Species–specific interaction networks

**Figure 4.**
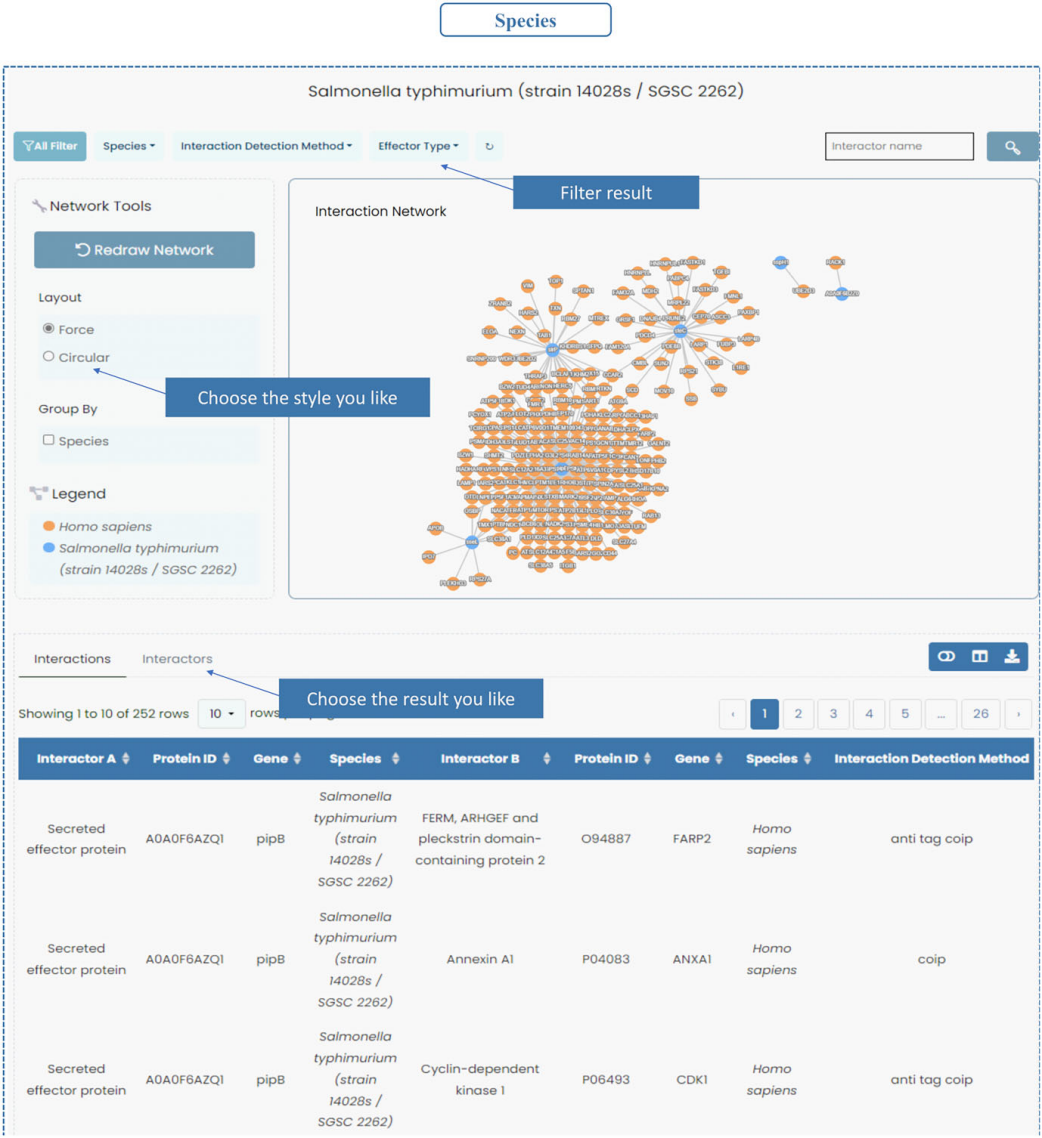
Species of InTxDB. InTxDB provides species-specific interaction networks, allowing users to explore bacterial effector–host interactions across different bacterial species. Users can filter results by species, interaction detection method, and effector type, with options to toggle between ‘Interactions’ and ‘Interactors’ for a more detailed analysis of the dataset.

InTxDB offers species-specific interaction networks, allowing users to explore bacterial effector–host interactions with enhanced filtering options. Users can refine results using the filter bar in the top left, selecting criteria such as species, interaction detection method, and effector type. Filtered entries can be toggled between ‘Interactions’ and ‘Interactors’, providing flexibility in data visualization. Each interaction or protein entry links to a detailed information page, enabling deeper insights into bacterial pathogenesis (Fig. 4).

### Tool

**Figure 5.**
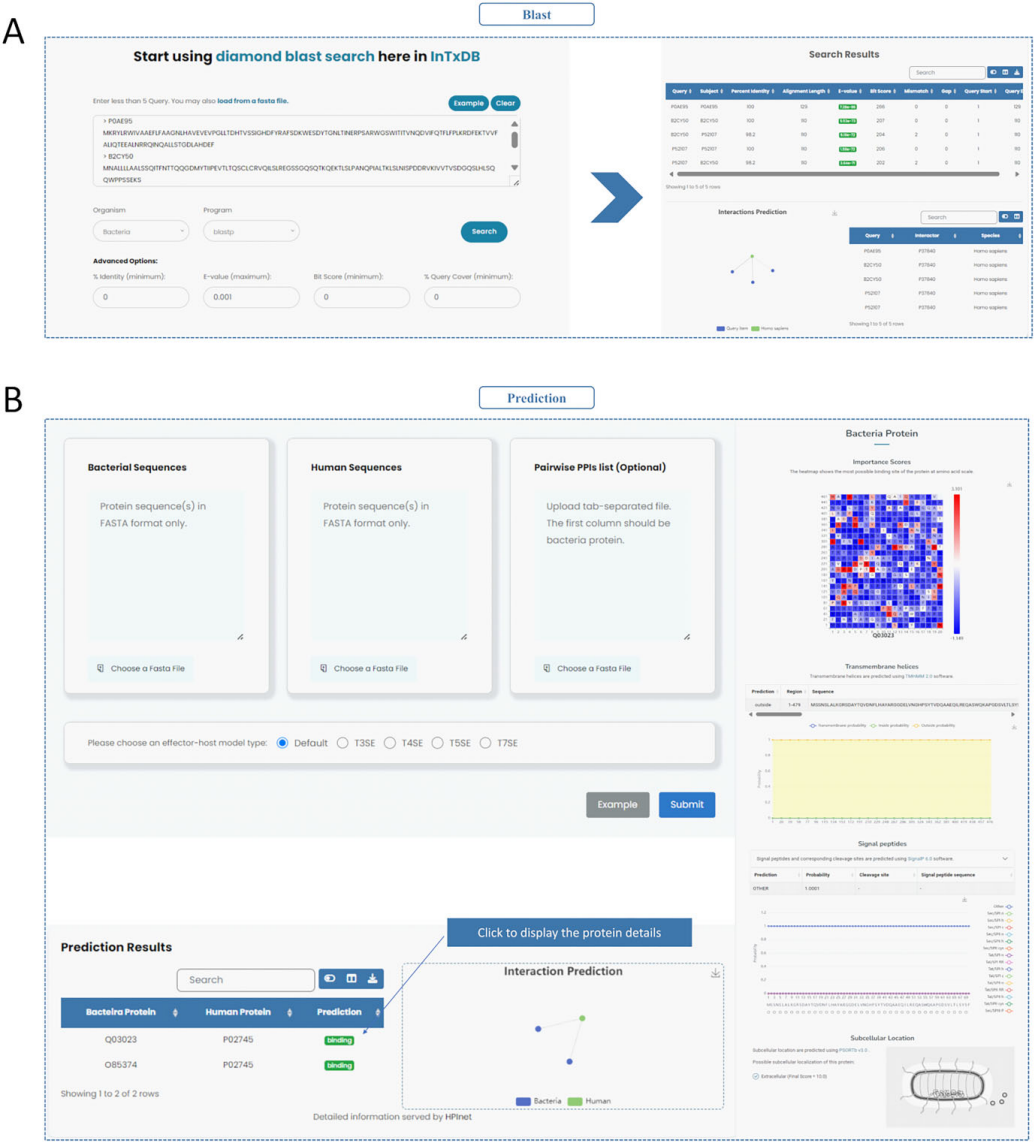
Tools of InTxDB. (A) Diamond tool: Provides sequence alignment capabilities with customizable parameters, allowing users to analyse and download alignment results. (B) HPInet Prediction tool: Predicts bacterial effector–host interactions based on sequence data or predefined PPI lists, generating network visualizations and residue importance heatmaps for deeper interaction analysis.

InTxDB provides two integrated data analysis tools to facilitate interaction prediction and sequence alignment. The Diamond [41] tool enables users to perform sequence alignment with customizable parameters and provides downloadable results (Fig. 5A). The HPInet Prediction tool [42] allows users to analyse interactions based on bacterial protein sequences, human protein sequences, or predefined PPI lists. The tool generates network structure visualizations and residue importance heatmaps, offering insights into key interaction sites (Fig. 5B). These tools enhance the functionality of InTxDB, supporting computational predictions and deeper analysis of bacterial effector–host interactions.

### Statistical insights

The Statistics section displays the distribution of bacterial species whose secretion effectors interact with host proteins, the classification of bacterial proteins, and the distribution of the four main subcellular localizations of human proteins that interact with effector proteins from different bacterial secretion systems. Additionally, functional enrichment analysis was performed on the human proteins, highlighting significantly overrepresented biological processes in Gene Ontology (GO), molecular pathways in KEGG, and regulatory processes in Reactome, thereby providing insights into their functional roles during pathogen–host interaction.

### Application in biological research

InTxDB serves as a valuable resource for biological research, such as investigating how LtxA, a leukotoxin secreted by *Aggregatibacter actinomycetemcomitans*, contributes to disease. By searching LtxA in InTxDB, users can identify its interaction with Integrin beta-2, a key immune cell receptor. Through PPI network analysis, it can be inferred that LtxA disrupts immune cell function, supported by previous findings [43]. Additionally, docking results help predict potential interaction targets, which may aid in developing novel biological therapies for conditions such as leukaemia, AIDS, and autoimmune disorders [44–46].

## Discussion

InTxDB provides a comprehensive resource for understanding the complex interactions between bacterial secretion effectors and host proteins, which are central to the pathogenesis of many bacterial infections. Unlike existing databases like IntAct [14] and PHISTO [12], which cover general host–pathogen interactions, InTxDB specifically focuses on bacterial secretion system effectors and their human targets. Additionally, it offers three-dimensional protein structures, docking models, and interaction site analyses, facilitating a deeper understanding of microbial virulence mechanisms and the identification of potential therapeutic targets.

Recent studies have emphasized the significance of bacterial effector proteins in manipulating host cellular processes to promote infection [47–50]. Among these, the type III secretion system (T3SS) effectors from *Salmonella* spp. play a pivotal role in hijacking host cell machinery to facilitate bacterial survival and replication [48–50]. For instance, the T3SS effector SopE2 reprograms macrophage metabolism, enabling *Salmonella typhimurium* to enhance intracellular replication and virulence [48]. Similar interactions have been studied for many other bacterial pathogens, demonstrating the widespread role of T3SS effectors in manipulating host cellular functions to promote infection [49]. InTxDB offers detailed data on these interactions, enabling researchers to explore not only the effectors involved but also their molecular mechanisms. The classification of bacterial proteins and their corresponding effector types can help researchers identify potential virulence factors and therapeutic targets [51, 52].

The HPInet prediction tool in InTxDB enhances the ability to predict PPI by leveraging known sequence data [42]. By integrating experimental data with predictive modelling, InTxDB enables users to make informed hypotheses about the roles of uncharacterized effectors or host proteins in bacterial infections.

Moreover, InTxDB provides subcellular localization data for human proteins interacting with bacterial effectors, which is crucial for understanding how these interactions influence cellular processes. Recent studies have demonstrated that the localization of host proteins plays a key role in bacterial infection strategies [53, 54]. For instance, the nuclear localization of the T4SS effector protein CBU0781 (AnkG) from *Coxiella burnetii* is essential for inhibiting apoptosis [54]. The anti-apoptotic activity of AnkG is regulated by p32-mediated intracellular transport, highlighting the importance of p32 localization in suppressing apoptosis during bacterial infection [54]. The subcellular localization statistics provided by InTxDB contribute to this growing body of knowledge. Understanding where these interactions occur within host cells is critical for designing therapeutic strategies that can block bacterial entry or disrupt bacterial manipulation of host cellular functions.

While the current version of InTxDB focuses exclusively on experimentally validated interactions to ensure high data reliability, we recognize the important potential of computational approaches to expand our understanding of host–pathogen interactions. In forthcoming updates, we plan to implement domain-based [30] or overlapping-fragment docking [31] to achieve more comprehensive structural coverage of these complexes. Additionally, we will introduce a dedicated module for high-confidence, computationally predicted interactions generated using state-of-the-art tools like AlphaFold3 [27] or AlphaFold-Multimer [55]. To further enhance biological relevance, predicted docking interfaces will be systematically analysed and mapped to known mutation sites, thereby enriching the interpretation of disease-associated variants. The database will continue to grow through the incorporation of newly published effector–host interaction datasets and expanded taxonomic coverage of gram-negative bacterial pathogens.

In conclusion, InTxDB serves as an essential resource for researchers in microbiology, immunology, and drug discovery. By providing access to high-quality datasets on bacterial effector–host protein interactions, along with powerful sequence alignment and predictive modelling tools, InTxDB accelerates research into bacterial pathogenesis and supports the discovery of novel therapeutic targets for combating bacterial infections.

## Conclusions

InTxDB provides a comprehensive and user-friendly platform for exploring the interactions between bacterial secretion effectors and host proteins, facilitating a deeper understanding of host–pathogen interactions. The database offers a robust suite of tools, including sequence alignment and prediction features such as Diamond [41] and HPInet [42]. These tools empower researchers to easily access, query, and analyse complex interaction data, uncovering valuable insights into the molecular mechanisms underlying bacterial pathogenesis. The inclusion of key statistics on bacterial species distribution, protein classification, and subcellular localization of human proteins further enriches the database, offering a holistic view of bacterial effector–host protein interactions. InTxDB is a valuable resource for the scientific community, enabling both basic and applied research in microbial pathogenesis, drug discovery, and therapeutic intervention strategies.

## Data Availability

The online version of InTxDB is freely accessible at http://bis.zju.edu.cn/InTxDB.
